# Soluble amyloid-β oligomers as synaptotoxins leading to cognitive impairment in Alzheimer’s disease

**DOI:** 10.3389/fncel.2015.00191

**Published:** 2015-05-26

**Authors:** Sergio T. Ferreira, Mychael V. Lourenco, Mauricio M. Oliveira, Fernanda G. De Felice

**Affiliations:** ^1^Institute of Biophysics Carlos Chagas Filho, Federal University of Rio de JaneiroRio de Janeiro, RJ, Brazil; ^2^Institute of Medical Biochemistry Leopoldo de Meis, Federal University of Rio de JaneiroRio de Janeiro, RJ, Brazil

**Keywords:** Alzheimer’s disease, amyloid-β oligomers, synapse failure, neuronal dysfunction, memory loss

## Abstract

Alzheimer’s disease (AD) is the most common form of dementia in the elderly, and affects millions of people worldwide. As the number of AD cases continues to increase in both developed and developing countries, finding therapies that effectively halt or reverse disease progression constitutes a major research and public health challenge. Since the identification of the amyloid-β peptide (Aβ) as the major component of the amyloid plaques that are characteristically found in AD brains, a major effort has aimed to determine whether and how Aβ leads to memory loss and cognitive impairment. A large body of evidence accumulated in the past 15 years supports a pivotal role of soluble Aβ oligomers (AβOs) in synapse failure and neuronal dysfunction in AD. Nonetheless, a number of basic questions, including the exact molecular composition of the synaptotoxic oligomers, the identity of the receptor(s) to which they bind, and the signaling pathways that ultimately lead to synapse failure, remain to be definitively answered. Here, we discuss recent advances that have illuminated our understanding of the chemical nature of the toxic species and the deleterious impact they have on synapses, and have culminated in the proposal of an Aβ oligomer hypothesis for Alzheimer’s pathogenesis. We also highlight outstanding questions and challenges in AD research that should be addressed to allow translation of research findings into effective AD therapies.

## Introduction

Alzheimer’s disease (AD) is a severe neurodegenerative disorder characterized by memory loss and progressive cognitive disability. It is the most prevalent form of dementia in the elderly, affecting more than 40 million people worldwide (Alzheimer’s Association, 2015). The number of AD cases is reportedly increasing and is expected to almost double by 2030 (Brodaty et al., 2011).

The current scenario is bleak: disease-modifying therapies are still not available, and existing strategies do not target potential causes of AD but rather downstream events in the disease. AD is a disease of complex etiology and, despite intense research efforts in the past few decades, no effective therapeutic approach has translated into clinical application. Meanwhile, costs associated with patient care and symptomatic therapeutics have escalated, making AD a major public health challenge in both developed and developing countries.

Understanding in molecular detail how AD develops represents a critical step towards determining which molecules and pathways should be targeted to efficiently halt or reverse neurological outcomes. AD is primarily a sporadic disorder: age is the main risk factor (LaFerla and Oddo, 2005) and fewer than 5% of the cases appear associated to familial inheritance (less than 1% linked to APP mutations; Tanzi and Bertram, 2001; Herrup, 2010). Thus, identification of toxins that accumulate with aging in the AD brain and the aberrant signaling pathways that lead to synapse dysfunction holds significant potential to pave the way for successful new therapeutic approaches.

Here, we focus on the discovery of soluble Aβ oligomers (AβOs) as toxins that accumulate in the AD brain and target synapses, and how these findings have led to significant advances in our understanding of mechanisms of memory impairment. We address the still unresolved issue of what is/are the receptor(s) to which AβOs bind at synapses, and further discuss recent evidence indicating that different oligomeric assemblies of Aβ may act together to orchestrate the complex pathogenesis of AD. Finally, we offer perspectives on how accumulated evidence from basic research might translate into clinical applications in AD.

## From Insoluble Amyloid Plaques to Soluble Aβ Oligomers

Following the initial description of the presence of amyloid plaques in the brain of a severely demented patient (Alzheimer, 1907; English-translated version in Alzheimer et al., 1995), eight decades passed before initial clues on their composition emerged. In 1984, Glenner and Wong isolated a novel peptide derived from AD-linked cerebrovascular pathology (Glenner and Wong, 1984). One year later, Masters and co-workers found that the same peptide, termed amyloid-β peptide (Aβ), is the principal component of amyloid plaque cores isolated from AD brains (Masters et al., 1985).

Aβ is a 4 kDa peptide (with 40- and 42-amino acid residue peptides as the predominant species) derived from proteolytic cleavage of a precursor protein termed amyloid precursor protein (APP; Kang et al., 1987; De Felice et al., 2004; Gralle and Ferreira, 2007). Aβ monomers readily aggregate in aqueous medium, giving rise to various types of assemblies including oligomers, protofibrils and amyloid fibrils. While AβOs are soluble and may spread throughout the brain, amyloid fibrils are larger and insoluble, and assemble into amyloid plaques, forming histological lesions that are characteristic of AD.

The idea that amyloid plaques might account for neurodegeneration (specifically, nerve cell death) and cognitive decline in AD became established in the so-called “amyloid cascade hypothesis”, supported by robust *in vitro* evidence pointing to a neurotoxic role of Aβ aggregates (e.g., Saitoh et al., 1989; Kowall et al., 1991; Pike et al., 1993; Lorenzo and Yankner, 1994), and put forward in an influential review by Hardy and Higgins (Hardy and Higgins, 1992). The central feature of that proposal was that deposition of aggregated Aβ into plaques would lead to neuronal death, ultimately causing dementia.

Nonetheless, as early as 1991, an intriguing report by Terry and colleagues (Terry et al., 1991) had demonstrated that synapse loss, rather than amyloid plaque load, provided the best pathological correlate of memory decline in AD. That notion was further supported by other studies that did not find direct correlation between plaque deposition and synapse/neuritic loss in postmortem analysis of AD brains (e.g., Masliah et al., 1990, 1993; Dickson et al., 1995). Further indicating a disconnect between plaque pathology and memory impairment, several groups reported that cognitive deficits in transgenic mouse models of AD appeared before plaque deposition or detection of insoluble amyloid aggregates in their brains (e.g., Hsia et al., 1999; Mucke et al., 2000; Westerman et al., 2002). Those observations puzzled researchers for years, and those transgenic animals were not regarded as bona-fide AD models because they exhibited memory impairment in the absence of plaque deposition. Nevertheless, we now understand that the build-up of soluble Aβ species, unknown and undetected at that time, likely underlies memory impairment in such transgenic mice (see below).

The apparent controversy on how amyloid-β toxicity correlated with cognitive decline and memory impairment in AD began to be resolved in 1998. In a seminal study, William L. Klein and colleagues demonstrated that, in addition to the well-known amyloid fibrils involved in plaque formation, Aβ spontaneously forms small, soluble oligomeric assemblies they termed Aβ-derived diffusible ligands (ADDLs). Although oligomeric Aβ had already been described at that time (Podlisny et al., 1995; Kuo et al., 1996; Roher et al., 1996), Lambert et al. (1998) first established that ADDLs could act as neurotoxins in the absence of amyloid fibrils or larger aggregates. ADDLs were shown to inhibit LTP and to promote cell death in a Fyn-dependent mechanism (Lambert et al., 1998). AβOs (more generally referred to as AβOs) are increased in AD brain extracts (Walsh et al., 2002; Gong et al., 2003; Kayed et al., 2003; Lacor et al., 2004; Townsend et al., 2006; Klyubin et al., 2008; Shankar et al., 2009; Xia et al., 2009) and can be detected using oligomer-sensitive antibodies (Lambert et al., 2001, 2007; Kayed et al., 2003; Rasool et al., 2013), but are not detected by conventional histopathological techniques, such as staining with Thioflavin S or Congo Red.

Soon after their identification, oligomers were incorporated into a revised version of the amyloid cascade hypothesis (Klein et al., 2001; Hardy and Selkoe, 2002; Selkoe, 2002a), which posited that soluble AβOs, rather than insoluble fibrils or plaques, would trigger synapse failure and memory impairment. As a corollary, AD would be a disorder of synapses (Selkoe, 2002b), and neuronal loss would only account for impaired brain function at final stages of the disease.

The emergence of AβOs as neurotoxins has considerably modified our understanding of disease mechanisms, not only in the specific case of Alzheimer’s but also of other neurodegenerative disorders in which oligomers formed by other proteins play causal roles (reviewed in Ferreira et al., 2007). Recently, this notion received support from promising results of a Phase 1 clinical trial of the high affinity antibody BIIB037 (Aducanumab), which binds both plaques and soluble oligomers (Clinical Trial ID # NCT01677572). BIIB037 has now been pushed into a Phase 3 trial and results are highly anticipated.

In the following sections, we review multiple synaptotoxic/neurotoxic effects of AβOs, which led to the proposal of an “Aβ oligomer hypothesis” of AD (Ferreira and Klein, 2011) in an attempt to better explain how oligomer accumulation in the brain leads to memory decline and cognitive dysfunction in AD.

## The Quest for AβO Receptors

AβOs appear to interact with a number of postsynaptic proteins assembled into a putative neuronal receptor complex that may include ionotropic and metabotropic glutamate receptors, the cellular prion protein (PrP^C^), neuroligin, the Wnt receptor Frizzled and insulin receptors, among other constituents (reviewed in Krafft and Klein, 2010; Ferreira and Klein, 2011; Viola and Klein, 2015). Many neurotoxic effects have been described as resulting from the interaction of AβOs with several receptors or co-receptors. We here provide a brief description of the main components of a putative receptor complex assembled by AβOs.

### Glutamate Receptors

Glutamate receptors have been closely implicated in the neurotoxicity of AβOs (Ferreira and Klein, 2011). Indeed, AβOs have been reported to interact with AMPA-type and NMDA-type ionotropic receptors (De Felice et al., 2007; Decker et al., 2010a; Zhao et al., 2010), as well as with type 5 metabotropic glutamate receptor (mGluR5; Renner et al., 2010; Um et al., 2013). AβOs appear to target synaptic sites in close proximity to AMPA receptors (AMPARs), and downregulation of AMPAR subunits prevents AβO binding to neurons (Zhao et al., 2010).

We have found that knockdown of NMDA receptors by a viral vector-mediated antisense approach (Decker et al., 2010a) or treatment of neurons with an antibody against the extracellular domain of the constitutive NR1 subunit of NMDA receptors (De Felice et al., 2007) block binding of AβOs to neuronal surface membranes. At the same time, however, presence of NMDA receptors on the neuronal surface is not sufficient for binding of AβOs to hippocampal neurons (Decker et al., 2010a). These results suggest that NMDA receptors are an integral component, likely an organizer of a receptor complex that binds oligomers at the neuronal surface.

Another protein that has received considerable recent attention as a putative oligomer receptor is mGluR5 (Renner et al., 2010; Hamilton et al., 2014; Hu et al., 2014). AβOs are capable of inducing mGluR5 clustering at the membrane, a mechanism responsible for increased calcium influx and neurotoxicity (Renner et al., 2010). Deletion of mGluR5 rescues memory deficits in APP/PS1 mice (Hamilton et al., 2014). Notably, both NMDARs and mGluR5 interact closely with PrP^C^ at the neuronal plasma membrane (Khosravani et al., 2008; Beraldo et al., 2011; Um et al., 2013), further suggesting that they may be part of a PrP^C^-containing oligomer receptor complex (see below).

### Prion Protein

Much attention has also been given to the role of PrP^C^ in oligomer binding. Since the original report that PrP^C^ binds AβOs and transduces neurotoxic signals (Laurén et al., 2009), there has been some controversy as to whether PrP^C^ is indeed necessary for oligomer binding and/or oligomer-induced brain dysfunction (Balducci et al., 2010; Kessels et al., 2010; Forloni and Balducci, 2011; Fluharty et al., 2013). Nonetheless, the possibility that PrP^C^ is a key component of an oligomer receptor complex remains attractive. Interestingly, exposure to AβOs recruits PrP^C^ molecules to the neuronal surface (Caetano et al., 2011), consistent with functional interaction between oligomers and PrP^C^ triggering signaling pathways to cause memory impairment (Gimbel et al., 2010; Um et al., 2012, 2013; Peters et al., 2015). Notably, AβO-induced Fyn activation, as previously demonstrated by Lambert et al. (1998), requires a receptor complex containing PrP^C^ and mGluR5 to cause neurotoxicity and memory impairment (Um et al., 2012, 2013). Furthermore, it was recently demonstrated that pharmacological inhibition of Fyn rescued cognitive performance in an AD mouse model (Kaufman et al., 2015).

### Frizzled Receptors

Wnt signaling has been shown to participate in many aspects of neuronal survival and function (Varela-Nallar and Inestrosa, 2013; Ríos et al., 2014). In the canonical pathway, Wnt binds to Frizzled receptors to stimulate β-catenin nuclear translocation and GSK-3β inhibition. We have reported that AβOs bind to a cysteine-rich domain of Frizzled that is directly involved in Wnt binding, thus impairing neuronal Wnt signaling (Magdesian et al., 2008) and resulting in altered expression of Wnt target genes, possibly including some related to synaptic plasticity mechanisms. Interestingly, Wnt has been found to be protective against the neurotoxicity of AβOs, reducing caspase-3 activation upon Frizzled-1 overexpression. Consistently, deletion of Frizzled-1 potentiated AβO effects (Chacón et al., 2008). Furthermore, activation of Wnt signaling counteracts behavioral impairments in AD mice (De Ferrari et al., 2003; Toledo and Inestrosa, 2010; Silva-Alvarez et al., 2013; Vargas et al., 2014). Thus, it is likely that recruitment of Frizzled to AβO receptor complexes precludes the activation of Wnt signaling and facilitates neuronal damage.

### Neuroligin

AβOs were reported to interact with the N-terminal tyrosine-rich domain of neuroligin-1. Moreover, *in vitro* purified N-terminal fragment of neuroligin stabilizes Aβ aggregates (Dinamarca et al., 2011). Intriguingly, neuroligin-1 can be cleaved under conditions of neuronal hyperexcitability, known to develop in AD (Sanchez et al., 2012; Vossel et al., 2013), in a process mediated by NMDARs (Suzuki et al., 2012). This could result in increased free extracellular N-terminal neuroligin fragments, thus facilitating AβO nucleation. Binding of AβOs to neuroligin could directly impact synapses, given that neuroligin associates with several partner proteins to maintain synapse structure and function. On the other hand, it was recently shown that amyloid fibrils suppress neuroligin expression to promote neurodegeneration (Bie et al., 2014). A combination of such events is likely to accelerate synapse damage and bran dysfunction in AD.

### Sigma-2/PGRMC1 Receptor

Sigma-2/PGRMC1 is the most recently described putative AβO receptor, and appears essential for oligomer neurotoxicity (Izzo et al., 2014a,b). AβOs increase total levels of this receptor in cultured hippocampal neurons (Izzo et al., 2014a), and both inhibition and knockdown of Sigma-2/PGRMC1 abolished AβO binding to synapses. Notably, a small molecule antagonist of Sigma-2/PGRMC1 receptors reversed AD memory impairment in the hAPPSwe/Ldn mouse model of AD (Izzo et al., 2014b). Future studies are warranted to understand how this protein might participate in the oligomer receptor complex, and which downstream signaling mechanisms promote neurotoxicity.

### Other Protein Receptors

In addition to the ones listed above, several other proteins have been reported to interact with AβOs. These include EphB2, p75 neurotrophin receptor (p75NTR), α7-nicotinic receptor, adrenergic receptors, receptor for advanced glycation end-products (RAGE), calcium channels, and less known components, such as LilRb2, formyl-peptideR2 and FcγRIIb (see Patel and Jhamandas, 2012; Viola and Klein, 2015, for reviews). Sortilin, in association with p75NTR, has also emerged as a candidate AβO binding protein (Takamura et al., 2012). Recently, AβOs have been reported to target ephrin A4 receptors to mediate synapse and cognitive dysfunction in AD mouse models (Fu et al., 2014; Overk and Masliah, 2014; Vargas et al., 2014).

### Non-Protein Receptors

Initial evidence provided by Lambert et al. (1998) indicated that AβO binding is trypsin-sensitive, suggesting that protein receptors are required for AβO interactions. Nevertheless, other types of molecules have been proposed to act as oligomer ligands. The ganglioside GM1 is a non-protein AβO ligand that has attracted considerable interest in recent years. AβOs target GM1-containing regions and lipid rafts (Hong et al., 2014), and bind directly to GM1 to promote neuronal damage (Williams et al., 2011; Calamai and Pavone, 2013). Furthermore, GM1-rich membrane domains are preferential sites for Aβ aggregation (Ogawa et al., 2011; Yamamoto et al., 2012, 2014), suggesting that GM1 could play an important role in the formation of seeds for Aβ oligomerization.

Some evidence also suggests that AβOs can bind directly to lipid membranes (Canale et al., 2013; Yates et al., 2013; Lee et al., 2014a), occasionally forming toxic pores (Arispe et al., 1993, 1996; Tofoleanu and Buchete, 2012; Zhang et al., 2012; Di Scala et al., 2014; Lee et al., 2014a), or serve as ligands to non-protein molecules within the plasma membrane, raising the possibility that direct neurotoxicity could be initiated by membrane interactions of sticky AβOs. A problem with this hypothesis, however, is that it fails to explain how intricate and specific cell-signaling effects could result from non-specific interactions of AβOs with the lipid bilayer at the plasma membrane.

### Direct and Indirect Interactions

Studies published during the past few years have resulted in a list of more than 15 proteins, some of which are reviewed above, that could act as oligomer receptors at the neuronal plasma membrane (Viola and Klein, 2015). Nonetheless, the nature of such interactions remains elusive in most cases. Few molecules, such as PrP^C^, neuroligin and Frizzled, have been shown to directly interact with AβOs, but the identity of the putative X receptor that mediates initial binding of oligomers is still unknown. Nevertheless, knockdown or depletion of several candidate receptors significantly reduce AβO binding to neurons or toxicity. This suggests that a multi-protein receptor complex is assembled by oligomers and mediates toxic signal transduction.

It is of interest that PrP^C^, known to physically bind AβOs, appears to function as a signaling platform at the neuronal plasma membrane, interacting with numerous partners (Linden et al., 2008). Thus, PrP^C^ may be well suited to act as an organizer of a multi-protein receptor complex responsible for the binding and transduction of the multiple neurotoxic effects of AβOs.

Initial interaction between AβOs and their immediate partners could be followed by recruitment of additional components to stabilize AβO binding. Thus, any single member of such a receptor complex would be required but not sufficient to maintain bound AβOs, as is the case for NMDAR (Decker et al., 2010a). Still, given the heterogeneity of oligomers populations, it is possible that oligomers of different sizes bind to different components within the receptor complex, increasing the complexity of signaling mechanisms.

## AβOs as Synaptotoxins in AD

Soluble Aβ (McLean et al., 1999) and AβOs accumulate in the brains and CSF of AD patients (Gong et al., 2003; Kayed et al., 2003; Lacor et al., 2004; Georganopoulou et al., 2005; Haes et al., 2005; Anker et al., 2009; Herskovits et al., 2013; Viola et al., 2015). Interestingly, oligomers are found at elevated levels and in association with synapses in the brains of demented AD individuals (Koffie et al., 2009; Bjorklund et al., 2012; Perez-Nievas et al., 2013), but they have not been detected at synapses in the brains of individuals who lacked *pre-mortem* cognitive impairment but whose brains presented typical AD neuropathology (Bjorklund et al., 2012). This suggests that the synaptic attack by AβOs could constitute the event leading to memory/cognitive dysfunction in AD. Immunofluorescence co-localization studies have revealed that AβOs target excitatory synapses (Lacor et al., 2004; Takahashi et al., 2004; Kokubo et al., 2005) and promote modifications in synapse structure and composition that disrupt synaptic function (e.g., Lacor et al., 2007; Shankar et al., 2007; Figueiredo et al., 2013). Although most current knowledge on the synaptic impact of AβOs comes from studies in cell culture or in rodent brains, these findings were recently corroborated in a novel non-human primate model of AD (Forny-Germano et al., 2014).

Although some evidence indicates that, at very low (picomolar) concentrations, AβOs may be important for proper synapse function (Puzzo et al., 2008, 2011; Garcia-Osta and Alberini, 2009; Lee et al., 2014b; Ricciarelli et al., 2014), increased levels of AβOs impair synaptic plasticity by inhibiting LTP (e.g., Lambert et al., 1998; Walsh et al., 2002; Klyubin et al., 2005, 2012; Gong et al., 2006; Hu et al., 2008; Jürgensen et al., 2011; An et al., 2013) and facilitating LTD (Wang et al., 2002; Li et al., 2009; Ma et al., 2013; Hu et al., 2014). Importantly, oligomers instigate memory impairment in rodents (Cleary et al., 2005; Lesné et al., 2006; Poling et al., 2008; Shankar et al., 2008; Figueiredo et al., 2013; Ledo et al., 2013; Lourenco et al., 2013), and blocking *de novo* AβO production acutely reverses synapse loss and memory impairment in APP transgenic mice in the absence of plaque load changes (Fowler et al., 2014).

Many studies have provided evidence (largely from immunofluorescence co-localization and/or co-immunoprecipitation experiments) supporting the notion that AβOs bind to excitatory synapses. However, recent studies have provided evidence that oligomers also bind to axons, albeit less robustly than to dendrites (Baleriola et al., 2014; Gan and Silverman, 2015), and that axonal binding may be, at least in part, responsible for the inhibition of fast axonal transport induced by AβOs (Decker et al., 2010b; Bomfim et al., 2012; Ramser et al., 2013). The specificity of synaptic targeting by AβOs thus deserves further examination.

Once bound to neurons, oligomers trigger multiple aberrant signaling pathways, including abnormal calcium signaling, oxidative stress, tau hyperphosphorylation, removal of plasticity-related receptors (e.g., AMPA and NMDA receptors) from synapses, impaired axonal transport, increased glutamate release from pre-synaptic terminals and elevated D-serine levels, endoplasmic reticulum (ER) stress and inflammation (Roselli et al., 2005; Rowan et al., 2005; Snyder et al., 2005; Hsieh et al., 2006; De Felice et al., 2007, 2008; Shankar et al., 2008; Decker et al., 2010a,b; Kurup et al., 2010; Saraiva et al., 2010; Wu et al., 2010; Brito-Moreira et al., 2011; Jürgensen et al., 2011; Miñano-Molina et al., 2011; Paula-Lima et al., 2011; Zhang et al., 2011; Bomfim et al., 2012; Yoon et al., 2012; Lourenco et al., 2013; Ma et al., 2013; Ramser et al., 2013; Madeira et al., 2015).

Tau hyperphosphorylation and development of neurofibrillary tangles (NFTs) are hallmarks of human AD pathology, and several studies have tried to unveil a potential relationship between amyloid and tau pathologies. Following the initial demonstration that AβOs induce tau hyperphosphorylation in cultured neurons (De Felice et al., 2008), other studies have extended those findings (Ma et al., 2009; Jin et al., 2011), and showed that tau is hyperphosphorylated in mice with increased AβO levels (Chabrier et al., 2012). The specificity of these events is supported by the fact that anti-Aβ antibodies block aberrant tau phosphorylation *in vitro* (De Felice et al., 2008; Jin et al., 2011) and *in vivo* (Serrano-Pozo et al., 2010). Moreover, tau hyperphosphorylation and NFT formation were recently demonstrated in a novel monkey model of AD generated by intracereboventricular (i.c.v.) injection of AβOs in cynomolgus monkeys (Forny-Germano et al., 2014). Recent evidence further suggests that amyloid pathology accelerates tau hyperphosphorylation and propagation *in vivo* (Pooler et al., 2015).

Tau can be phosphorylated by several kinases that are overactive in AD brains (e.g., GSK-3β, Cdk5, Cdc2, PKR, JNK and other MAPKs; reviewed by Mandelkow and Mandelkow, 2012). Significant evidence suggests that AβO-mediated tau phosphorylation leads to cytoskeleton disruption and synaptic loss (Zempel et al., 2010, 2013; Zempel and Mandelkow, 2012). Zempel and collaborators have shown that AβOs induce tau mis-sorting, a phenomenon in which axonal tau moves to dendrites in hyperphosphorylated form, disrupting cytoskeletal organization and axonal transport (Zempel et al., 2010), ultimately instigating neuronal collapse. In accord with this notion, AβOs were shown to impair activity-dependent tau re-localization in neurons (Frandemiche et al., 2014).

Tau pathology has further been shown to cause brain oxidative and ER stress in animal models of AD (Dias-Santagata et al., 2007; Abisambra et al., 2013; Frost et al., 2014) and, of clinical relevance, Aβ-linked cortical volume loss only develops in the presence of p-tau, further connecting amyloid to tau pathology (Desikan et al., 2011). Deletion of tau was further shown to protect hippocampal cultures from the toxicity of fibrillar Aβ and APP23 mice from developing cognitive impairment mediated by Fyn (Ittner et al., 2010).

Successive failures of anti-amyloid therapeutics in promoting cognitive benefits in AD patients has raised the possibility that tau might be a better target for therapeutic development, given its involvement in disease mechanisms. Nonetheless, despite the toxic effects aberrant tau may cause, considerable evidence now indicates that AβOs act upstream of tau to promote neuronal dysfunction. In fact, some aspects of toxicity, such as defective BDNF axonal transport, are not necessarily dependent on tau (Ramser et al., 2013; Takach et al., 2015). Thus, specific targeting of AβOs could be the key for developing effective approaches to prevent both tau-dependent and tau-independent defects in AD.

Of considerable interest are findings that AβOs impair neuronal insulin signaling (Townsend et al., 2007; Zhao et al., 2008; De Felice et al., 2009; Bomfim et al., 2012), providing an explanation at the molecular/cellular level for the connection between AD and diabetes revealed by clinical/epidemiological studies (reviewed in De Felice, 2013). Insulin receptors are widely distributed in the brain (Zhao and Alkon, 2001; Zhao et al., 2004). In addition to its regulatory actions in energy metabolism, insulin has been found to play important roles in synaptic plasticity (Chiu et al., 2008), learning and memory (reviewed in Fernandez and Torres-Alemán, 2012). Following the discovery that AβOs trigger removal of insulin receptors from neuronal plasma membranes (Zhao et al., 2008; De Felice et al., 2009), we have shown that oligomers instigate activation of neuronal stress kinases, including c-Jun N-terminal kinase (JNK), IκB kinase (IKK), and double-stranded RNA-dependent kinase (PKR), resulting in phosphorylation of the insulin receptor substrate 1 (IRS-1) at inhibitory serine residues, and blocking downstream signaling in the insulin pathway (Bomfim et al., 2012). This mechanism is analogous to what takes place in peripheral tissue, leading to insulin resistance in type II diabetes (Hotamisligil et al., 1996; Gregor and Hotamisligil, 2011). Combined removal of insulin receptors from the plasma membrane and serine phosphorylation of IRS-1 effectively blocks neuronal insulin signaling (as verified in AD brain tissue; Talbot et al., 2012), thus impeding plasticity-related actions of insulin at synapses.

Additional AβO-triggered mechanisms of synaptic dysfunction have been elucidated in the past few years. Of particular interest, elevated eIF2α phosphorylation (eIF2α-P) was recently correlated to synapse loss induced by AβOs (Lourenco et al., 2013), consistent with the finding that eIF2α kinases are overactive in brain areas responsible for memory processes in AD (Chang et al., 2002; Hoozemans et al., 2005; Yoon et al., 2012; Ma et al., 2013) and with the known role of eIF2α-P in attenuating global protein translation (Buffington et al., 2014). Importantly, suppression or inhibition of eIF2α kinases improves learning and memory in a physiological context (Costa-Mattioli et al., 2005, 2007; Zhu et al., 2011; Di Prisco et al., 2014) and alleviates memory deficits in AD models (Lourenco et al., 2013; Ma et al., 2013). Synapse damage and loss in AD might further be mediated by a mechanism involving ER stress and activation of the unfolded protein response (UPR), which comprises eIF2α-P as a key player. In fact, elevated levels of UPR markers are found in AD models (Lourenco et al., 2013; Ma et al., 2013), as well as in postmortem AD brains (Chang et al., 2002; Hoozemans et al., 2005, 2009; Chafekar et al., 2007; Yoon et al., 2012). Aberrant activation of the UPR and, in particular, eIF2α-P appears dependent on elevated TNF-α levels and TNFR1 activity, pointing to a central role of neuroinflammation in synapse loss and memory impairment in AD models (Lourenco et al., 2013). Blocking TNFR1 signaling or alleviating brain ER stress through the use of chemical chaperones abrogate AβO-induced cognitive impairment (Lourenco et al., 2013), in accordance with findings in AD transgenic models (He et al., 2007; McAlpine et al., 2009; Ricobaraza et al., 2009, 2012).

## Differential Impact of Low- and High-Molecular Weight AβOs on Synapses

As noted above, AβOs trigger multiple neurotoxic/synaptotoxic mechanisms *in vitro* and *in vivo*, resulting in impaired cognition. Most of the current knowledge on the neuronal impact of AβOs comes from studies using cell-derived or synthetic oligomer preparations comprising a mixture of species ranging from low-n oligomers (dimers, trimers, tetramers) to higher-order assemblies typically ranging in MW from 50 to 150 kDa.

The complete molecular pathway that leads to Aβ aggregation in its multiple oligomeric forms is still unclear. Although still controversial, significant evidence points to the roles of seed species, including Aβ dimers, trimers and Aβ*56, in the build up of amyloid in the brain (Bitan et al., 2003; Hellstrand et al., 2010; O’Nuallain et al., 2010; Garai and Frieden, 2013; Tsigelny et al., 2014). Aβ monomers rapidly assemble into oligomers that might or not consecutively turn into higher-order aggregates (Rangachari et al., 2007; Velasco et al., 2012), and a stepwise aggregation process has been proposed to generate AβOs in the AD brain (Velasco et al., 2012). Whatever the precise pathway of aggregation of Aβ, high molecular weight oligomers are increased in the CSF of AD patients (Fukumoto et al., 2010) and in AD brain (Gong et al., 2003). In APP/PS1 mice, both low and high molecular weight oligomers are increased in the CSF (Takeda et al., 2013).

Given their molecular heterogeneity, it is possible that different oligomeric species trigger diverse effects in the brain by recruiting distinct signaling mechanisms. This is in line with the complex spectrum of molecular/behavioral outcomes seen in a multifaceted disorder such as AD. Indeed, specific Aβ assemblies (e.g., dimers, trimers and Aβ*56) have been reported to mediate distinct pathological effects in the diseased brain (e.g., Lesné et al., 2006, 2013; Reed et al., 2011).

Recently, we reported that low molecular weight (LMW; dimers-tetramers) and high molecular weight (HMW; ranging from ~50 to ~150 kDa) AβOs have different impacts on synapses, despite the fact that both LMW and HMW oligomers cause memory deficits in mice (Figueiredo et al., 2013). While LMW oligomers alter synaptic composition (revealed by decreased synaptophysin immunoreactivity), HMW oligomers induced neuronal oxidative stress *via* activation of NMDA receptors. Notably, mice i.c.v. injected with HMW oligomers had recovered from memory impairment 14 days post-injection, while memory damage persisted in mice injected with LMW oligomers (Figueiredo et al., 2013). It is, thus, likely that persistent memory impairment triggered by LMW oligomers results from synapse loss, while the reversible memory loss induced by HMW oligomers may result from aberrant activation of NMDARs, which could be blocked by memantine (Figueiredo et al., 2013). The differential impact on synapses is further supported by a recent report showing that low-n and high-n oligomers have differential binding affinities for neurons, with a population that ranges from decamers to hexadecamers exhibiting preferential synapse targeting (Velasco et al., 2012). HMW species have been recently found to significantly interact with PrP^C^ in AD brains (Dohler et al., 2014), suggesting that PrP^C^ might be important for transducing signals of defined AβO assemblies.

Low-molecular weight oligomers (SDS-stable dimers, trimers and tetramers) were shown to cause synapse loss (Shankar et al., 2007) and acutely impair synaptic plasticity (Chen and Glabe, 2006), with a particularly toxic role for trimers (Townsend et al., 2006). LMW oligomers were also reported to cause tau phosphorylation, cytoskeleton destabilization and cell loss *in vivo* (Davis et al., 2011; Brouillette et al., 2012).

Elevated Aβ dimers are detected in an age-dependent manner in AD brain (Lesné et al., 2013) and in the brains of J20 APP transgenic mice (Shankar et al., 2009). Dimers isolated from AD brain or CSF impair synapse homeostasis *in vivo* (Klyubin et al., 2008; Shankar et al., 2008) and their neutralization by monoclonal antibodies corrects synaptic plasticity defects (Klyubin et al., 2008; O’Nuallain et al., 2011). Interestingly, levels of Aβ dimers are increased in AD patients independently of disease progression, suggesting that once a certain level of dimers is attained in the brain, further accumulation is not necessary for disease progression (McDonald et al., 2012). Trimers, on the other hand, correlate with increased *in vitro* neurotoxicity (Hung et al., 2008) and are thought to act as seeds for generation of high-molecular weight oligomers (Matsumura et al., 2011).

Nonetheless, the actions of dimers/trimers on synapses and cognition remain somewhat controversial as recent reports found that preparations of Aβ dimers did not impact LTP in hippocampal slices (O’Malley et al., 2014) and transgenic brain-extracted trimers did not impact memory even when injected at high concentrations in rats (Reed et al., 2011), findings that are in harmony with *in vivo* evidence from Lesné et al. (2006). Still, the possibility remains that Aβ dimers/trimers are converted into more toxic species in the presence of additional factors and biophysical conditions that are specific to aged human brains.

Additional low-n species, such as tetramers, have also been proposed to play a distinct role in AD etiology (Bernstein et al., 2009). In a clinical study, Aβ pentamers and decamers were found to correlate with alterations in the cholinergic system, a prominent feature of AD (Bao et al., 2012). Notably, a very recent study has indicated that HMW oligomers, but not LMW oligomers, accumulate in forebrain cholinergic regions in AD, consistent with the deleterious role of AβOs on cholinergic neurotransmission (Baker-Nigh et al., 2015).

Despite our current knowledge of a myriad of detrimental actions of AβOs in neurons, most cellular/synaptic effects of AβOs are still orphan in terms of the identity of the specific oligomeric assemblies involved. In part, and as recently pointed out (Benilova et al., 2012), this is due to the heterogeneity of oligomer preparations used in different studies, which depends on factors including source of the peptide (i.e., synthetic vs. “naturally secreted” by cell lines transfected with human APP, or brain-extracted oligomers), presence of chemical/post-translational modifications in the peptide (e.g., pyroglutamylation, methione oxidation, tyrosine cross-linking), the specific protocol used to prepare oligomers, conditions of storage and aging of oligomer preparations, etc. Intriguingly, for example, recombinant Aβ_42_ was reported to aggregate faster and to be more neurotoxic than its synthetic version (Finder et al., 2010), and different assembles from synthetic or cell-derived oligomers were found to vary in terms of cognitive outcomes in rats (Reed et al., 2011). This exemplifies some of the complexities involved in comparing results from different groups and using different experimental approaches, and has led to considerable controversy and conflict in the literature. Progress in this area would greatly benefit from identification of the specific assemblies (minimally, in terms of low vs. high molecular weight oligomers) involved in specific effects of AβOs at synapses.

## AβOs and their Interactions with Glial Cells

Despite being widely considered neurotoxins that attack synapses with high affinity, evidence has now accumulated that AβOs also elicit responses from glial cells, namely astrocytes and microglia. Indeed, it is plausible that glial responses to AβOs actively participate in neurotoxicity and brain dysfunction. Still, whether the same receptor composition as in neurons is required for AβO binding to glial cells remain unknown.

Astrocytes are known to internalize AβOs (Nielsen et al., 2010), and a recent report suggested that even low concentrations of AβOs induce calcium influx and generation of reactive oxygen species (ROS) in astrocytes, in a mechanism that could even be triggered by a single oligomer particle (Narayan et al., 2014). Through ERK signaling, AβOs were also shown to impair astrocytic metabolism (Tarczyluk et al., 2015) and insulin signaling (Zhang et al., 2015), likely contributing to the observed glial reactivity upon exposure to AβOs (Ledo et al., 2013). Impaired insulin signaling and increased astrogliosis may further facilitate the production of pro-inflammatory cytokines in the brain, as suggested by several reports (Carrero et al., 2012; Ledo et al., 2013; Lourenco et al., 2013). Additional studies are required to further elucidate how AβOs interact with astrocytes, likely disturbing their physiology and propagating neurotoxic signals in the brain.

AβOs also target microglia to promote TNF-α and IL-1β release (Ledo et al., 2013). TNF-α, in turn, stimulates stress kinase activity in neurons, resulting in impaired insulin signaling and cognition in mice (Bomfim et al., 2012; Lourenco et al., 2013). Cytokine profiling in AD brains suggests that microglial cells exhibit a phenotype often referred to as M1-like, characterized by augmented expression of pro-inflammatory cytokines, including IL-1β and TNF-α (Saijo and Glass, 2011).

Still, it is not clear whether pro-inflammatory responses are a result of direct or indirect effects of oligomers on microglia. A number of reports indicate that microglial receptors such as CD36, RAGE, scavenger receptor B-1 (SCARB-1), scavenger receptor A1 (SCARA-1) and TLRs 2 and 4, could directly mediate AβO actions (reviewed by Saijo and Glass, 2011). On the other hand, AβO neurotoxicity and activation of aberrant signaling pathways in neurons may result in secretion of alarmin-like molecules to induce microglial activation. Indeed, a mechanism of this type has been described involving CX3CR1, a chemokine receptor essential for neuron-microglia communication (Fuhrmann et al., 2010).

A complete picture of the contributions of each group of cytokines and chemokines to AβO toxicity is still not available. For example, knock out of interleukin-10 (IL-10), an anti-inflammatory cytokine, ameliorated cognitive impairment in APP/PS1 mice, consistent with enhanced IL-10 signaling in AD brains (Guillot-Sestier et al., 2015). Administration of IL-10 in APP/PS1 mice reduced Aβ phagocytosis, contrary to observations in IL-10-depleted animals (Guillot-Sestier et al., 2015). Suppression of interleukin receptor-associated kinase 4 (IRAK4), a downstream effector of TLR activity, resulted in improved clearance and reduced Aβ levels in APP/PS1 mice (Cameron et al., 2012). Similarly, overexpression of interleukin-1β, a pro-inflammatory cytokine, reduced amyloid plaque burden in 3 × Tg mice, although tau pathology was worsened (Ghosh et al., 2013).

Upon activation, microglia act as potent phagocytic cells, and this may be important as a defense mechanism to prevent early Aβ deposition. However, significant accumulation of AβOs appears to impair phagocytic activity of microglia. Stimulation of the PPARy/RXRa heterodimer by selective agonists intensifies Aβ phagocytosis by microglial cells and improves memory performance in APP/PS1 mice (Yamanaka et al., 2012). This appears to be mediated by CD36, a molecule that has reduced expression in APP/PS1 mice, in a mechanism that could be mediated by TNF-α (Hickman et al., 2008).

In conclusion, AβOs impact astrocytic and microglial physiology, eliciting toxic pro-inflammatory responses, including TNF-α release, contributing to brain damage in AD. In addition, it is possible that exposure to increased AβO levels leads to impairments in mechanisms essential for proper brain function, such as neuromodulator release and phagocytosis. Glial loss-of-function instigated by AβOs could impact neurotransmission and synapse homeostasis, a possibility supported by recent reports that glial gene expression is essential for memory and may be disrupted in AD (Matsuno et al., 2015; Sekar et al., 2015). Thus, glial-mediated effects initiated by AβOs in the brain may occur by increased release of toxic molecules, impairment of essential glial functions in neuromodulation, or both.

## Current Challenges in AD Research

Several questions pertaining to the mechanisms of AD pathology have been resolved after three decades of research centered on the Aβ peptide. Yet, many major questions still demand answers, particularly regarding the specific synaptotoxic/neurotoxic outcomes of the accumulation of low and high molecular weight AβOs in the AD brain. Further, the precise mechanisms by which oligomers trigger neurotoxicity are not fully understood (Benilova and De Strooper, 2011). One possibility is that AβOs bind to a multi-protein receptor complex at the neuronal surface, and this triggers parallel aberrant activation of several signaling pathways that result in synapse failure and neuronal damage (Krafft and Klein, 2010; Ferreira and Klein, 2011). Another possibility is that different-sized oligomers bind to different receptors, triggering selective neurotoxic pathways. In this regard, we recently obtained evidence that high and low molecular weight oligomers do not compete for the same receptor at neuronal surfaces (Achurra and Ferreira, unpublished data). It may well be, therefore, that the neuronal impact of low molecular weight oligomers (which specifically results in persistent structural damage to synapses revealed by synaptophysin loss; Figueiredo et al., 2013) differs substantially in terms of the receptors and activated pathways from the impact of high molecular weight oligomers, which appears to be centrally mediated by NMDARs and can be blocked by the NMDAR channel blocker memantine (Figueiredo et al., 2013).

Another major gap in our current knowledge comprises the interaction of oligomers with glial cells (notably, astrocytes and microglia), how such interaction may lead to potentially damaging aberrant activation of glial cells, and the potential role of glial cells in oligomer clearance. In particular, nothing is known about the potentially different interactions of LMW and HMW oligomers with glial cells.

Lastly, we feel that major efforts should be directed at generating improved AD models. Most of the currently available animal models are based on introduction of familial AD-linked mutations in rodents, resulting in abnormally elevated Aβ levels and deposition, and AD-like pathology. While such transgenic models have revealed many important aspects of AD progression, they can at best be considered models for familial (early onset) cases of AD, which represent no more than 5% of the total disease burden (LaFerla and Oddo, 2005). Moreover, as previously pointed out (Selkoe, 2011), transgenic mouse models of AD do not recapitulate the full spectrum of AD pathology. For example, transgenic mouse models of AD lack the NFTs that are a hallmark of human pathology, and induction of tangle formation in mice depends on expression of a mutant form of tau that is not found in AD patients (LaFerla and Green, 2012). Most transgenic mouse models also fail to exhibit neuronal death as observed in later states of AD. It is noteworthy that a recently developed AD rat model engineered to express mutant human APP and PS1 genes was reported to develop cognitive impairment accompanied by AβO production, tau pathology and neuronal loss (Cohen et al., 2013), and a mouse model developed by LaFerla and co-workers expressing Arctic APP at low levels presented enhanced AβO formation and wild-type tau pathology (Chabrier et al., 2012).

Also noteworthy is that APP transgenic mice express different Aβ assemblies (from monomers to plaques), making it difficult to assign specific pathogenic roles and mechanisms to each type of Aβ aggregate. An attempt to overcome this hurdle was the development of a mouse model for AD that markedly accumulates AβOs and exhibits synapse and memory loss in the absence of amyloid fibrils and plaque deposition (Tomiyama et al., 2010). Similar cognitive impairment was found in another transgenic model at pre-plaque ages (Ferretti et al., 2011).

The inability to recapitulate cardinal features of the neuropathology of AD, on top of the essential differences between rodent and human brains in terms of the neural circuitry and architecture, may explain why a large number of therapeutic approaches that were validated in transgenic mouse models of AD have disappointingly failed in human trials.

Recent approaches have been undertaken to develop alternative experimental models that might be relevant to sporadic AD and to human pathology. We have shown that human adult cortical tissue slices can be maintained in culture for several days, allowing investigation of the effects of AβOs in *ex-vivo* human brain tissue (Sebollela et al., 2012). This approach has allowed an initial investigation of oligomer-induced changes in gene expression in human brain tissue, and, among other findings, showed that synaptophysin is markedly down-regulated, at both mRNA and protein levels, in human brain tissue exposed to AβOs (Sebollela et al., 2012). Clearly, access to surgical human brain tissue represents a bottleneck for wider dissemination of this experimental model. Nonetheless, the relevance of results obtained in intact human neural tissue should not be underestimated in considering the translational potential of this model for development of novel therapeutic approaches in AD.

In terms of *in vivo* modeling of sporadic AD, two recent initiatives are worth mentioning. First, i.c.v. injection of AβOs in mice triggers aberrant signaling pathways (Bomfim et al., 2012; Lourenco et al., 2013) that culminate with memory loss and behavioral alterations that recapitulate clinical observations in AD patients (Figueiredo et al., 2013; Ledo et al., 2013; Lourenco et al., 2013). The relative ease of implementation and robustness of this approach make it an attractive alternative for pathway elucidation as well as for screening of novel treatments or preventative schemes. It should be kept in mind, however, that this represents an acute model, as neuropathology and cognitive deficits are detected between one day and a few weeks following a single i.c.v. injection of AβOs, much faster than the course of several months or years for disease progression in transgenic mice or humans, respectively. It is possible, therefore, that the acute impact of AβOs in mice brains triggers signaling pathways that might not play significant roles in the chronic nature of human disease. Moreover, mechanistic studies in rodents might not fully recapitulate human disease, as rodents do not spontaneously develop AD features such as Aβ deposition and accelerated cognitive decline. Therefore, the creation of novel animal models that better approximate human AD seems warranted.

Development of a novel monkey model of AD was recently reported (Forny-Germano et al., 2014). I.c.v. infusion of AβOs in cynomolgus monkeys triggered brain pathology that was quite reminiscent of neuropathological alterations that are characteristic of AD, including tau hyperphosphorylation, formation of neurofibrillary inclusions, and synapse loss. The similarity in pathological features triggered by oligomers and the overall close proximity between macaque and human brains suggests that this novel model may be very relevant for studies aimed to understand in greater detail the mechanisms of pathogenesis instigated by AβOs, and to improve translation of experimental results into therapeutic approaches. Such therapeutic interventions could be useful not only in AD but also, as recently proposed (Morrison and Baxter, 2014), to maintain synapse health in age-related cognitive impairment and to prevent the onset of dementia in the elderly.

Finally, induced pluripotent stem cells (iPSCs) derived from AD patient fibroblasts (Yagi et al., 2011; Israel et al., 2012; Kondo et al., 2013) have been generated in the past 4 years. AD-derived cells present increased tau phosphorylation (Israel et al., 2012) and Aβ oligomer accumulation (Kondo et al., 2013), and similar findings were described in a 3D culture of neural stem cells derived from familial AD cases (Choi et al., 2014). Generation of sporadic AD-derived iPSCs is of special interest as it might provide insight into how non-familial AD cases initiate and develop. As technology for iPSC generation develops and costs are reduced, it is anticipated that such cells will become a valuable resource for high-content screening of potential neuroprotective or disease-modifying approaches.

## Concluding Remarks

**Figure 1:**
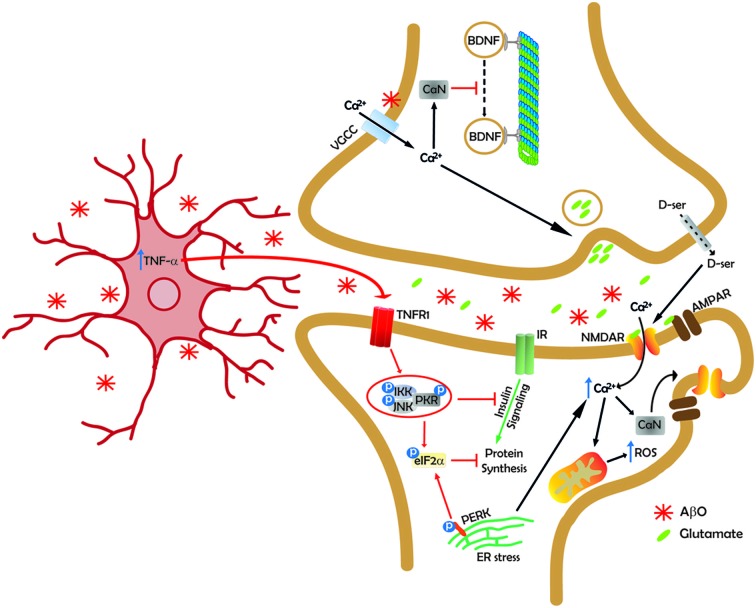
**Synapse under siege**. A simplified scheme illustrating some of the mechanisms by which AβOs (AβOs; represented as red asterisks) impact synapse plasticity and function. At the pre-synaptic terminal, AβOs inhibit microtubule-based fast axonal transport (FAT) in a tau-independent manner (Decker et al., 2010b; Ramser et al., 2013; Gan and Silverman, 2015; Takach et al., 2015), resulting in reduced or interrupted transport of various cargoes, including brain-derived neurotrophic factor (BDNF), to synapses. FAT inhibition may involve physical or functional interaction of AβOs with axonal voltage-gated calcium channels (VGCC), calcium influx into the axon and activation of calcineurin (CaN). Deregulated calcium levels also triggers increased pre-synaptic glutamate release (Brito-Moreira et al., 2011). Release of the NMDA receptor (NMDAR) co-agonist, D-serine, is also increased in neurons exposed to AβOs (Brito-Moreira et al., 2011; Madeira et al., 2015), resulting in elevated basal excitatory tonus. At the dendritic spine, AβOs aberrantly activate NMDARs, triggering increased calcium influx and increased production of reactive oxygen species (ROS; De Felice et al., 2007; Decker et al., 2010a; Saraiva et al., 2010; Paula-Lima et al., 2011), likely mediated by mitochondrial dysfunction. NMDAR-mediated calcium influx causes ryanodyne receptor-mediated calcium release from endoplasmic reticulum (ER) stores (Paula-Lima et al., 2011). Elevated calcium levels at the spine activate CaN to dephosphorylate NMDARs and AMPARs at specific phosphoepitopes, causing their removal from synapses and internalization (Snyder et al., 2005; Hsieh et al., 2006; Jürgensen et al., 2011). CaN activation has also been implicated in Aβ-induced spine loss (Wu et al., 2010; not represented in the scheme for simplicity). Calcium-dependent activation of calcium/calmodulin-dependent kinase II (CaMKII) and casein kinase II (CKII) also appears to mediate removal of NMDARs from synapses (De Felice et al., 2009; not represented in the current scheme). AβOs block insulin signaling by instigating removal of insulin receptors (IRs) from the neuronal plasma membrane (De Felice et al., 2009; not represented here), inhibiting IR autophosphorylation (Zhao et al., 2008; not represented), and by inhibiting IR-mediated tyrosine phosphorylation of the insulin receptor substrate-1 (IRS-1; Bomfim et al., 2012; not represented). AβOs instigate brain microgliosis and increased microglial production/secretion of TNF-α (Ledo et al., 2013; unpublished results). Aberrant TNF-α signaling activates cell stress kinases (c-Jun N-terminal kinase, JNK; IκB kinase, IKK; double-stranded DNA-dependent protein kinase, PKR; Bomfim et al., 2012; Lourenco et al., 2013), resulting in serine phosphorylation and further inhibition of IRS-1. PKR also phosphorylates eukaryotic initiation factor 2 α (eIF2α; Lourenco et al., 2013). eIF2α is further phosphorylated by PKR-related ER resident kinase (PERK), a kinase that executes one of the three branches of the unfolded protein response (UPR) that is activated by ER stress in neurons exposed to AβOs (Lourenco et al., 2013; Ma et al., 2013). Insulin signaling inhibition and eIF2α-P lead to blockade of protein synthesis, essential for synaptic plasticity (Buffington et al., 2014). Additional mechanisms (not included here) thought to further contribute to synapse failure include deregulation of the actin cytoskeleton at spines (Lacor et al., 2004; Davis et al., 2011), loss of pre- and post-synaptic proteins (e.g., synaptophysin, PSD-95; Roselli et al., 2005; Sebollela et al., 2012; Figueiredo et al., 2013), Fyn kinase-mediated surface removal of NMDARs and spine loss (Um et al., 2012), among others.

In conclusion, compelling evidence supports the notion that AβOs are the proximal toxins that cause synapse failure and, ultimately, memory loss in AD. Although questions remain to be answered, e.g., concerning the identity (or identities) of the neuronal receptor(s) to which oligomers bind and the size/nature of the most synaptotoxic species, we now know a great deal about aberrant signaling pathways and mechanisms by which oligomers affect neuronal physiology and, specifically, synapse function (Figure 1). Recent efforts have also aimed to develop experimental models that more closely recapitulate the human disease and allow for detailed testing of novel potentially disease-modifying therapeutics. Together, such developments offer reasons for optimism in the quest towards development of effective approaches to prevent or rescue synapse damage in AD.

## Conflict of Interest Statement

The authors declare that the research was conducted in the absence of any commercial or financial relationships that could be construed as a potential conflict of interest.
